# Supplementary material for: Investigating the distribution of antibiotic resistance genes in relation to bacterial, fungal, and functional diversity in a hay field
Source: Microbiol Spectr. 2026 Apr 10;14(5):e03232-25. doi: 10.1128/spectrum.03232-25 (PMC13141933; doi:10.1128/spectrum.03232-25)
Supplement: Supplemental Figures — Figures S1 to S8. [file spectrum.03232-25-s0001.docx]

Investigating the distribution of antibiotic resistance genes in relation to bacterial, fungal, and functional diversity in a hay field

Carolina Oliveira de Santana^1,2^, Pieter Spealman^3^, Conrad Vispo^4^, David Gresham^3^, Sage Saccomanno^5^, Christopher N. LaFratta^5^, Swapan S. Jain^5^, Robert S. Dungan^6^,

& Gabriel G. Perron^2,3*#^

^1^ Department of Biological Sciences, State University of Feira de Santana, Feira de Santana, Bahia, Brazil.

^2^ Bard Center for Environmental Sciences and Humanities, Bard College, Annandale-On-Hudson, NY, 12504.

^3^ Center for Genomics and Systems Biology, New York University, New York, NY 10004, USA.

^4^ Hawthorne Valley Farmscape Ecology Program, Hawthorne Valley Association, Ghent, NY, 12075, USA.

^5^ Chemistry and Biochemistry Program. Bard College, Annandanle-On-Hudson, NY 12504.

6 USDA-ARS, Northwest Irrigation & Soils Research Laboratory, Kimberly, Idaho 83341, USA

*#Corresponding author:*

Department of Biology

Reem-Kayden Center for Science and Computation

Bard College

30 Campus Road, Annandale-on-Hudson, NY, 12571

Tel. 845-752-2334; email. [gperron@bard.edu](mailto:gperron@bard.edu)

# Supplemental Materials


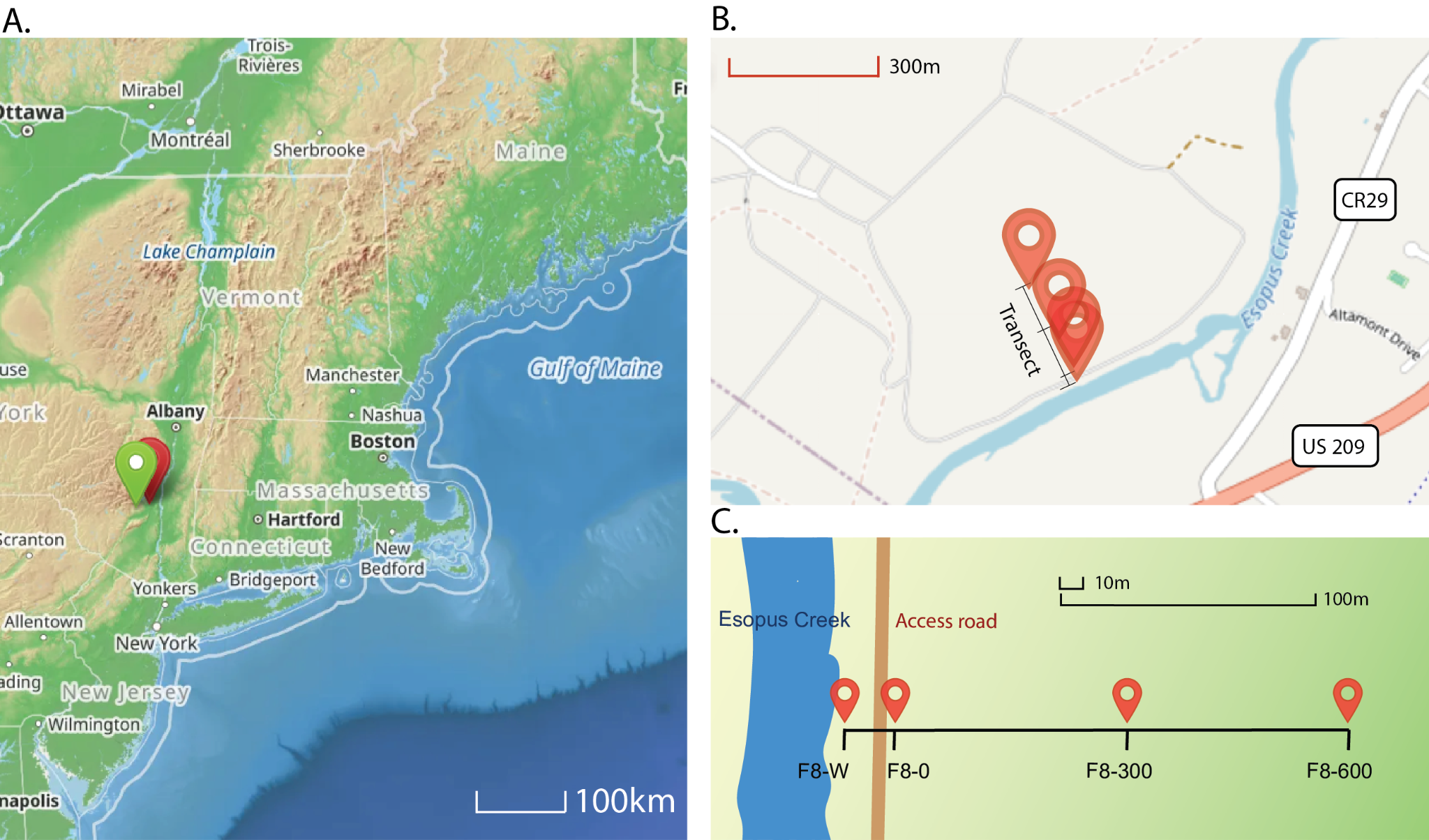


### **Figure S1. Labelled map of sampling sites**

Environmental sampling of a hay field transect in Ulster County, New York, USA (**A, B**). Four sample sites were selected and sequenced F8-W (41.90927,-74.08550); F8-0 (41.90945,-74.08553); F8-300 (41.91020,-74.08600); F8-600 (41.91089,-74.08651); (**C**). Figures A,B generated using openstreetmap.org

### **
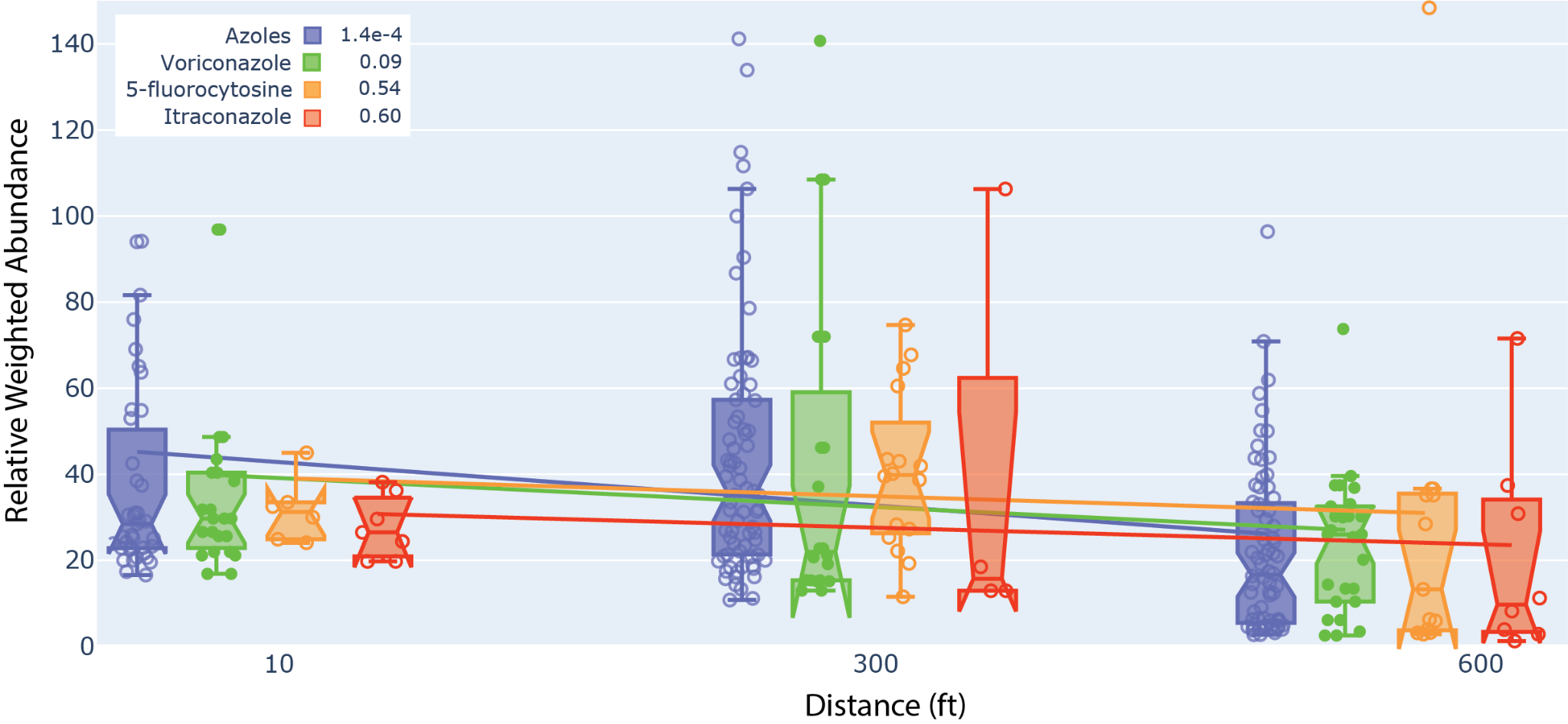
**

### **Figure S2. Relative antifungal resistance gene abundances relative to transect distances**

We identified 320 likely antifungal resistance genes (E-value < 0.05) across seven categories from the proteins predicted from the WGS data. Fluconazole, Micafungin, and Caspofungin were removed from consideration for being observed less than 10 times across all samples. We then used linear regression to determine if any of the remaining categories had significant changes in abundance relative to distance. Of the remaining classes: Azoles, Voriconazole, 5-fluorocytosine, and Itraconazole all had slightly negative trends as distance increased but only the broad category of Azoles was significant with a p-value of less than 0.05.

*
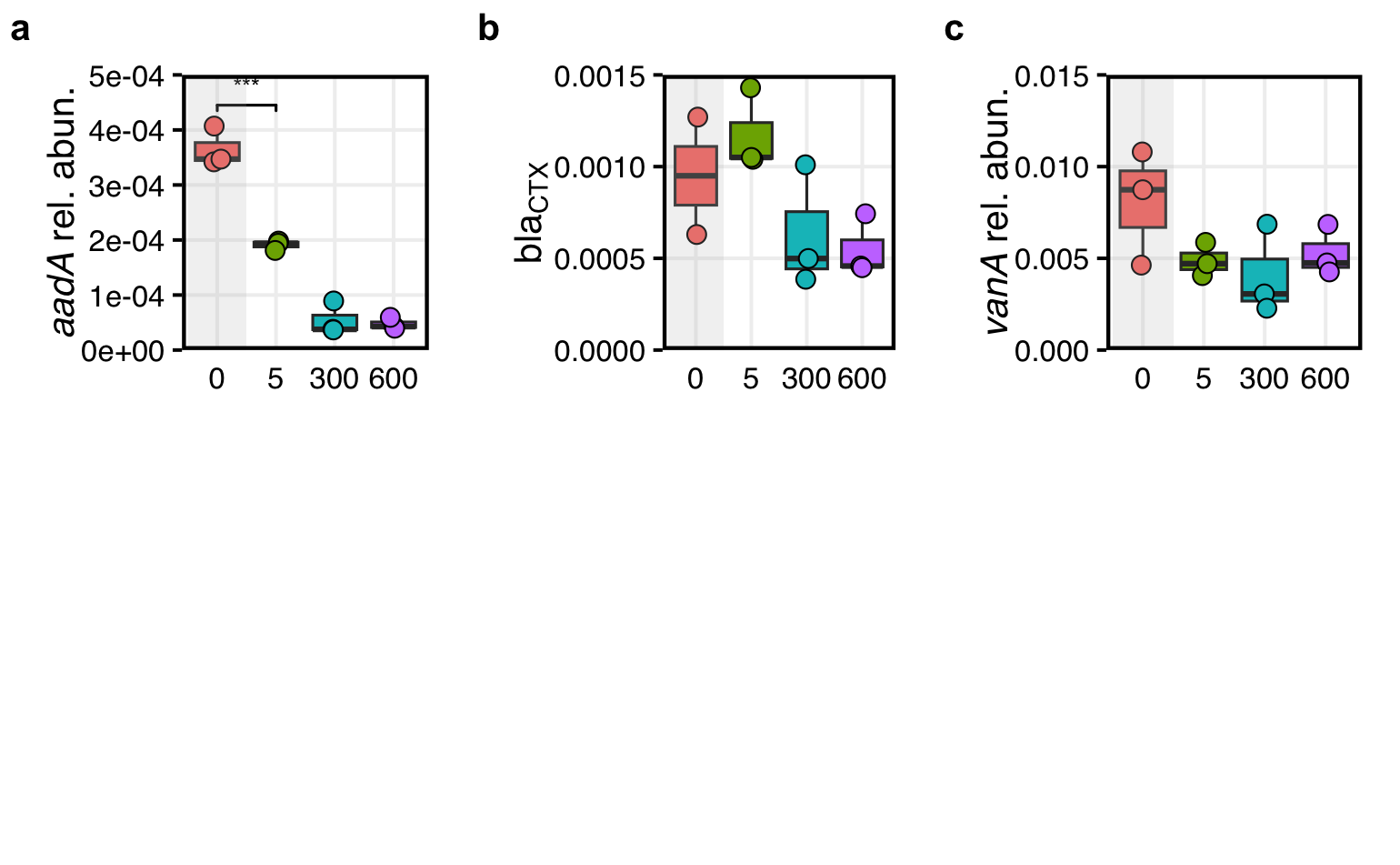
*

### **Figure S3. Relative abundance of three antibiotic resistance genes along the F8 agricultural transect**. Relative abundance for antibiotic resistance genes **a**) *aadA*, **b**) *blaCTX*, and **c**) *vanA* was estimated by dividing the gene copy number by the copy number of *16S rRNA* gene identified in each sample using quantitative PCR. We used ANOVA to compare r.a. between site F8-W and the transect sites while we used linear regression analyses to assess the trend observed in the transect, i.e., excluding F8-W. We found a significant decrease in relative abundance for *aadA* and *blaCTX* and no statistically significant trend for *vanA*. Each dot represents an individual replicate collected at each site and whiskers represent quartiles.


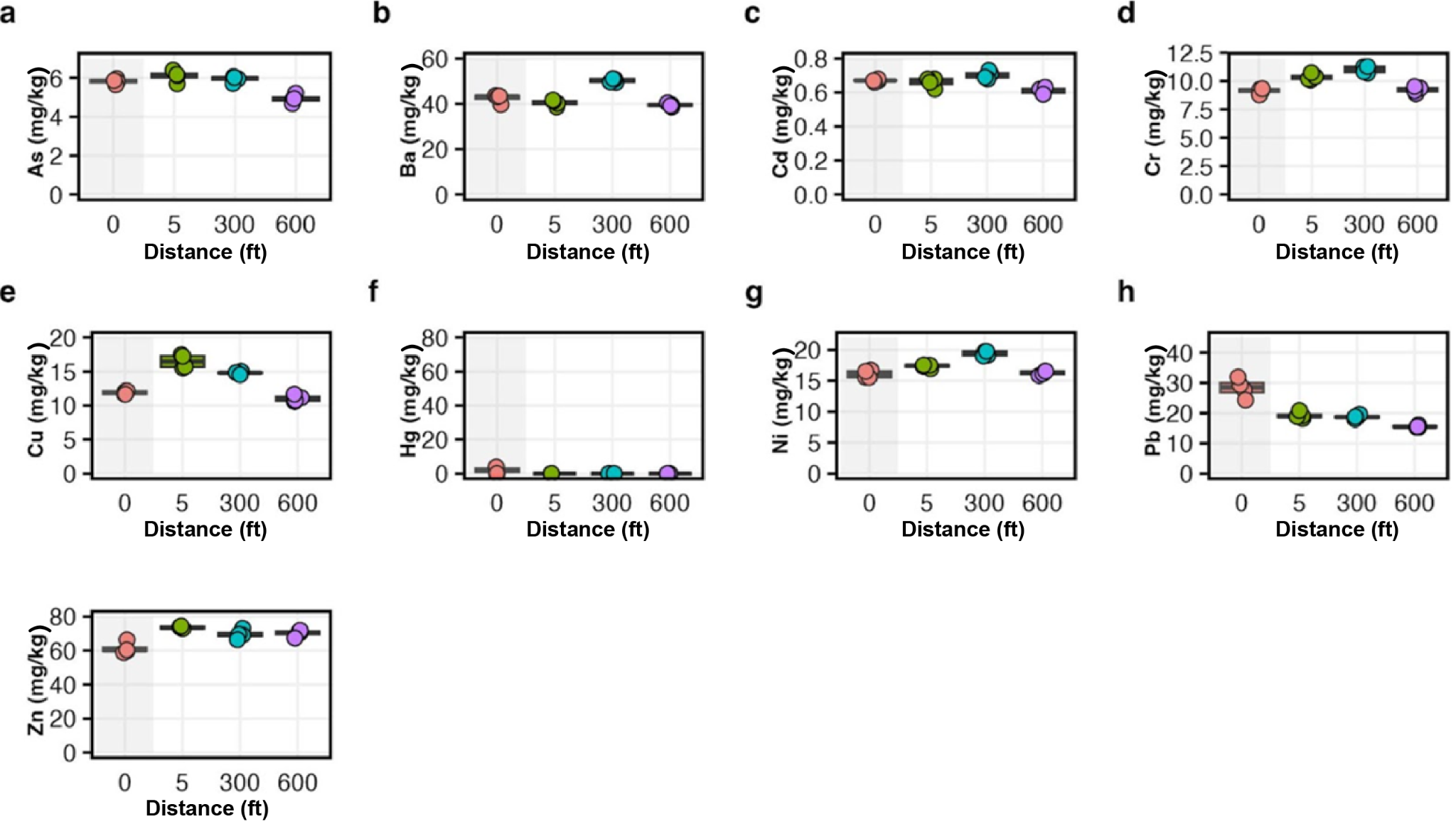


| **Figure S4. Concentration of nine heavy metals along the F8 agricultural transect**. Concentrations (mg/kg) trends over distance (ft) were estimated using two methods for **a**) Arsenic (As), **b**) Barium (Ba), **c**) Cadmium (Ca), **d**) Chromium (Cr), **e**) Copper (Cu), **f**) Mercury (Hg) **g**) Nickel (Ni), **h**) Lead (Pb), and **i**) Zinc (Zn). We used ANOVA to compare concentrations between site F8-W and the transect sites while we used linear regression analyses to assess the trend observed in the transect, i.e., excluding F8-W. Each dot represents an individual replicate collected at each site. |
| --- |


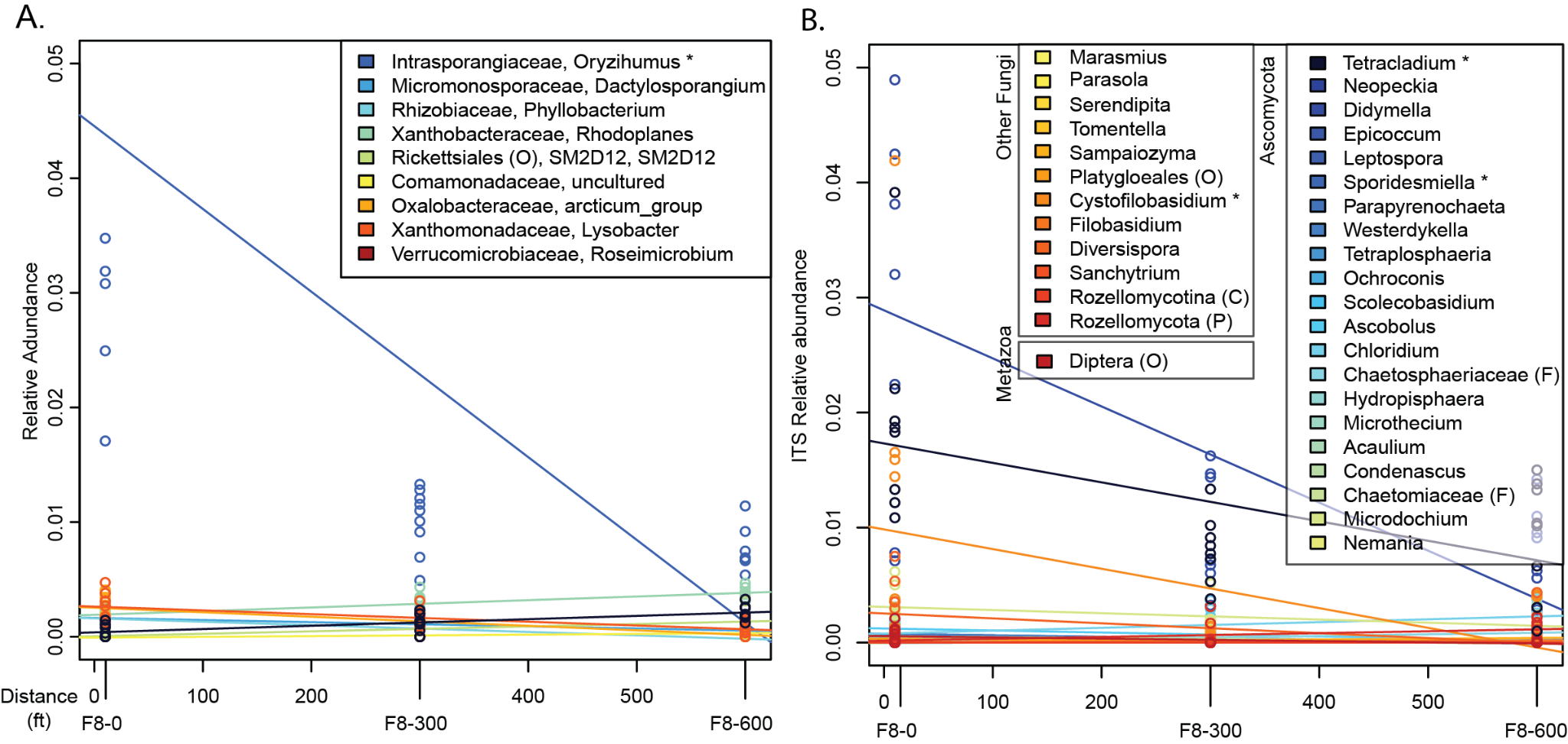


### **Figure S5. Abundance of genera as a function of distance**. (A) We used a linear model of the relative abundance of bacterial genera as a function of distance and only found one genera, *Oryzihumus,* with a relative abundance greater than 1% that significantly changed as a function of distance. (B) Similarly for fungal genera, we found 3 genera with relative abundances greater than 1% significantly changed over distance from the water; the Ascomycota *Sporidesmiella* and *Tetracladium*, and the Basidiomycota *Cystofilobasidium* (B).


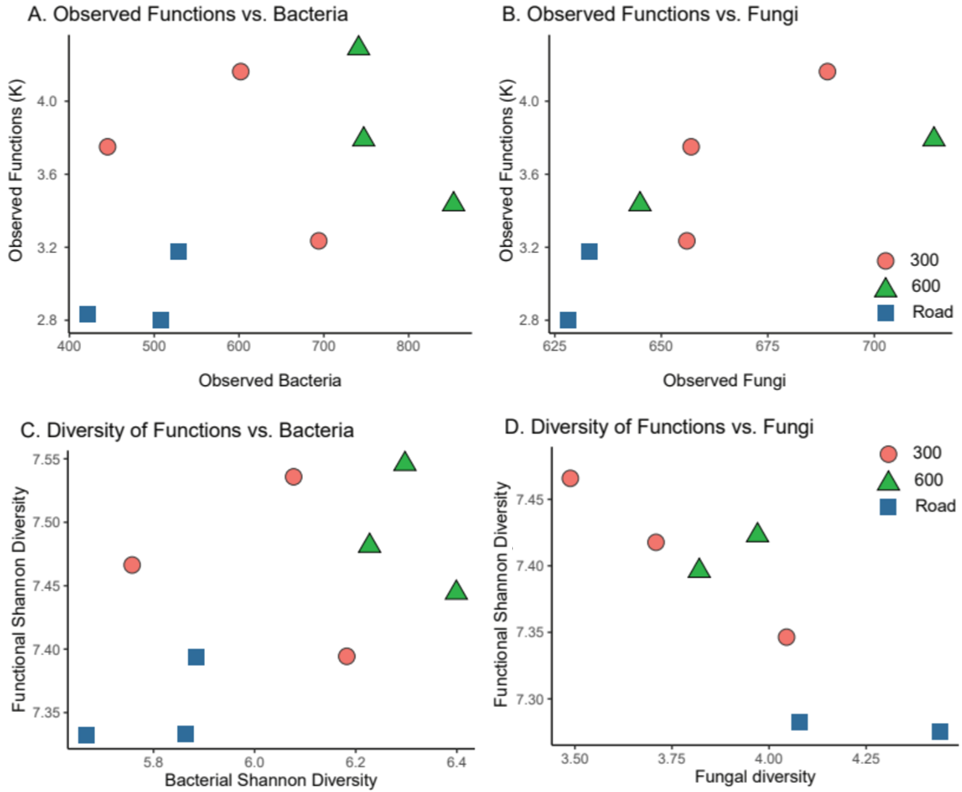


| **Figure S6. Correlation between diversity of functions identified and microbial communities.** (**A**) We find that the total number of observed predicted functions did not significantly increase with the number of observed bacterial ASVs (*F*_(1,6)_ = 3.23; *R*^2^ = 0.56, *P* = 0.12) (**C**) nor that the diversity (H) of functional genes correlated with bacterial diversity (*F*_(1,6)_ = 0.09; *R*^2^ = 0.46, *P* = 0.77). (**B**) we found that the total number of functions did not significantly correlate with the number of observed fungal ASVs (*F*_(1,4)_ = 3.52; *R*^2^ = 0.45, *P* = 0.13), but that (**D**) functional diversity measured as Shannon Diversity Index, was negatively correlated with fungal diversity (*F*_(2,2)_ = 316.12; *R*^2^ = 0.99, *P* = 0.003) suggesting that fungal diversity could play an important role in shaping functional diversity. |
| --- |


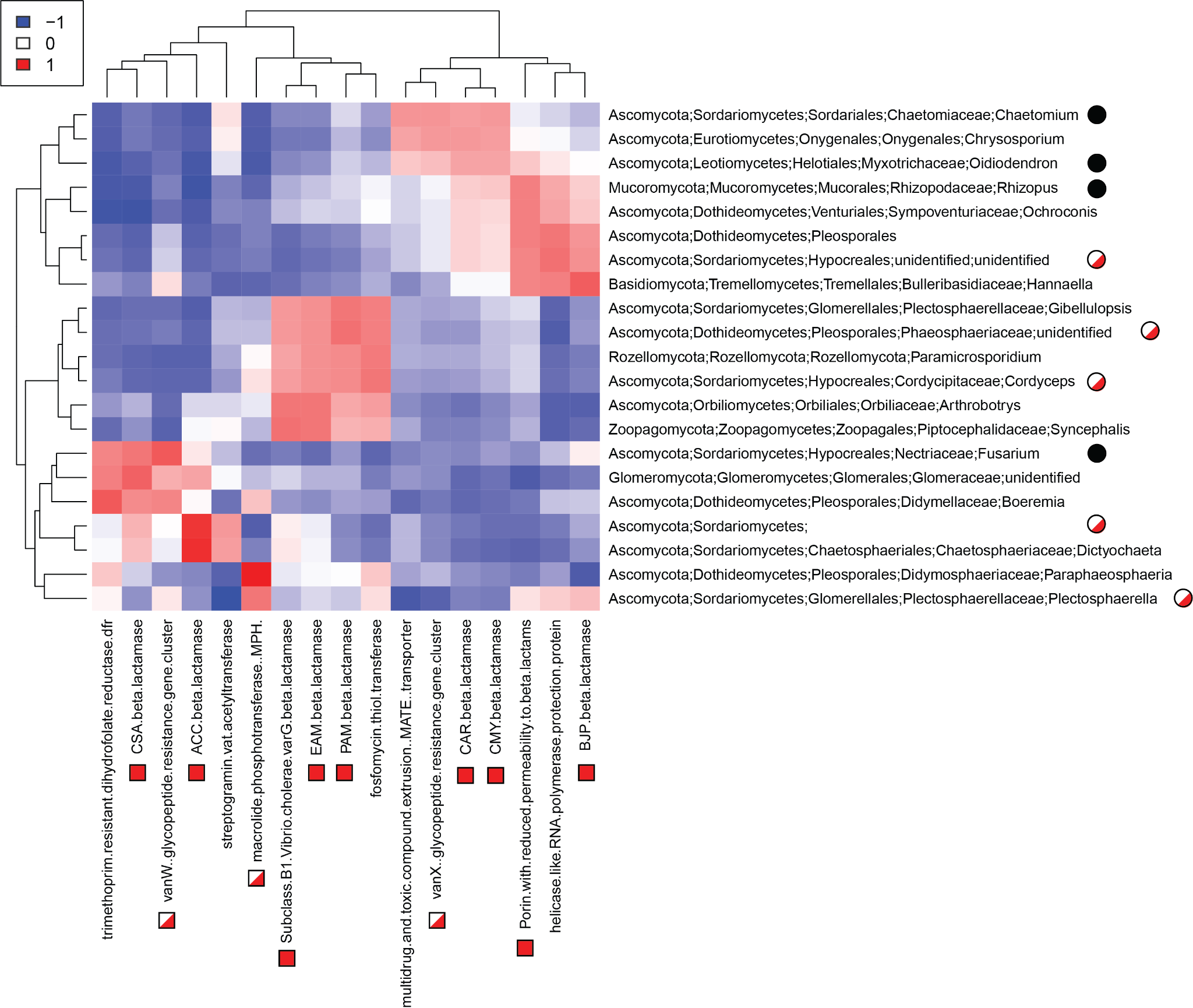


### **Figure S7. Correlation between ARG drug class family identified and fungal genera.**

Using linear regression we can calculate the correlation between fungal taxa abundance and the family of drug an ARG provides resistance to. Using this method we find that 9 out of 17 drug families that are significantly correlated with fungal taxa provide resistance to beta-lactam (red squares), a class of antibiotics that includes penicillin and cephalosporin and are commonly produced by fungi. An additional 3 families provide resistance to glycopeptides and macrolides, two classes of antibiotic that can also be produced by fungi (half-filled square).
 Importantly, beta-lactam production in fungi is probably the result of horizontal gene transfer from bacteria [(107)](https://paperpile.com/c/KBkyi5/Q85F) no genera identified is known to produce beta-lactam, although some are known to produce beta-lactam precursors or lactam-like antibiotics (half-filled circles). Conversely, several fungal taxa (black circles) such as *Fusarium* are known to produce beta-lactamases [(67)](https://paperpile.com/c/KBkyi5/wmtz). Suggesting that this correlation may be caused, not by the production of beta-lactam by fungi but fungi with resistance to beta-lactam produced by bacteria.


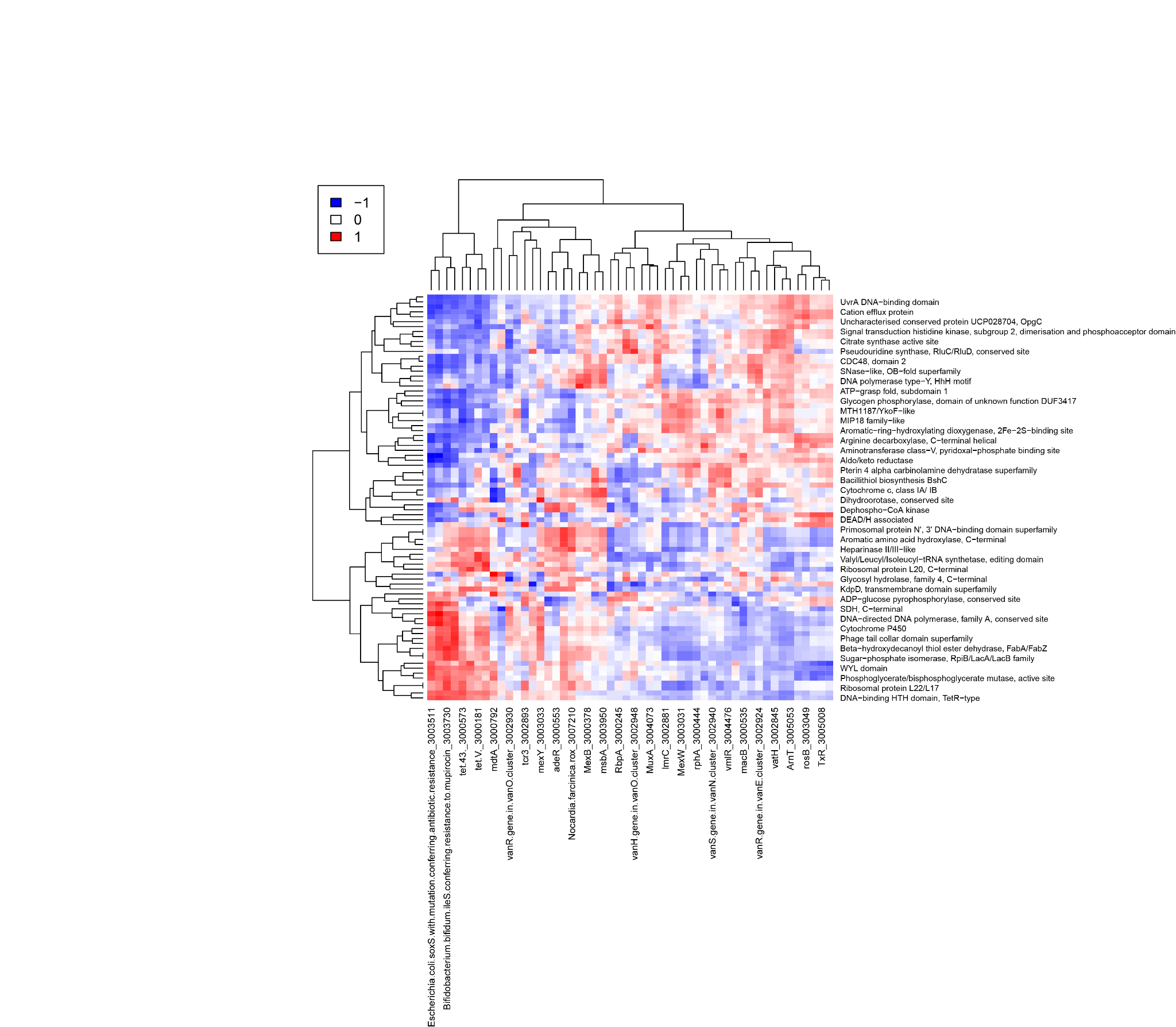


### **Figure S8. Correlation between antibiotic resistance genes and predicted gene functions.** Heatmap shows the significantly correlated (R-value) of the relative abundances of functions and ARGs estimated using linear regression, i.e., Benjamin-Hochberg adj-*P*s <= 0.01. Each function and ARG must be observed in a minimum of six samples to be included. Four clinical ARGs are present, macB, MexB, MexW, MexY.

### **Supplemental Tables**:

#### **Table S1.** Tab delimited file. Contains sequencing run quality control numbers for amplicon sequencing runs generated by denoising using DADA2.

#### **Table S2.** Tab delimited file. Contains QIIME2 Feature IDs with taxonomic assignments and confidence scores.

#### **Table S3.** Tab delimited file. Contains omnibus and pairwise alpha-diversity tests (Evenness, Faith-PD, Shannon) and beta-diversity tests (Jaccard, Bray-Curtis, Unweighted UniFrac, Weighted UniFrac) for both ITS (rarefaction = 350K) and 16S (rarefaction = 10K) runs.

#### **Table S4.** Tab delimited file. Contains all pairwise Bray-Curtis dissimilarity (ie. percentage difference) for all 16S and ITS samples.

#### **Table S5.** Condition for antibiotic resistance gene qPCR.

#### **Table S6.** Analysis of functions significantly correlated with distance, their Interpro ID, broad category, and association with antibiotic resistance or mobile genetic elements, if any.
